# Induction of procalcitonin in liver transplant patients treated with anti-thymocyte globulin

**DOI:** 10.1186/cc6202

**Published:** 2007-12-18

**Authors:** Roman Zazula, Miroslav Prucha, Tomas Tyll, Eva Kieslichova

**Affiliations:** 1 Department of Anesthesiology and Intensive Care, Charles University in Prague, the First Faculty of Medicine and Thomayer's Faculty Hospital, Videnska 800, 140 59 Prague, Czech Republic; 2 Department of Clinical Biochemistry, Hematology and Immunology, Hospital Na Homolce, Roentgenova 2, 150 30 Prague, Czech Republic; 3 Department of Anesthesiology and Intensive Care, Institute for Experimental and Clinical Medicine, Videnska 1958/9, 140 21 Prague, Czech Republic

## Abstract

**Introduction:**

The aim of this study was to compare the early postoperative kinetics of procalcitonin (PCT) and C-reactive protein (CRP) serum levels in patients undergoing orthotopic liver transplantation (OLTx) with different immunosuppressive regimens.

**Methods:**

PCT and CRP serum concentrations were measured in a group of 28 OLTx recipients before induction of anesthesia, at 4 and 8 hours following graft reperfusion, and daily until postoperative day 4. The same parameters were determined in 12 patients undergoing liver resection without conjunctive immunosuppressive therapy. Summary data are expressed as medians and ranges. Two-tailed nonparametric tests were performed and considered significant at *p *values of less than 0.05.

**Results:**

The highest serum levels of PCT (median 3.0 ng/mL, minimum 1.4 ng/mL, maximum 13.9 ng/mL) were found in patients after OLTx without ATG therapy, on postoperative day 1. In patients with ATG administration, PCT levels were highly increased on postoperative day 1 (median 53.0 ng/mL, minimum 7.9 ng/mL, maximum 249.1 ng/mL). Thereafter, PCT values continuously decreased independently of further ATG administration in both groups of patients. No evidence of infection was present in either group. In 12 patients undergoing liver resection, peak serum PCT levels did not exceed 3.6 ng/mL. CRP serum levels in a group of patients with and without ATG therapy increased significantly on postoperative day 1, followed by a decrease. The highest levels of CRP were found in patients after liver resection on postoperative day 2 and decreased thereafter.

**Conclusion:**

ATG administration to patients with OLTx is associated with an increase in serum PCT levels, with peak values on postoperative day 1, and this was in the absence of any evidence of infection. The results of this study indicate that ATG immunosuppressive therapy is a stimulus for the synthesis of PCT.

## Introduction

At the beginning of the '90s, it was discovered that elevated levels of serum procalcitonin (PCT) were closely related to the infectious etiology of systemic inflammatory response. Its role as a marker of infectious inflammation was reported repeatedly, and today PCT is assessed as a sensitive and specific marker of severe bacterial inflammation [1,2].

The last meta-analysis established that PCT is a more sensitive and specific parameter for the evidence of systemic bacterial infection than C-reactive protein (CRP) [2]. An increased PCT level over the course of the first 24 hours is an independent predictor of all-cause mortality in a 90-day follow-up period [3].

In patients undergoing organ transplantations, markers allowing the differentiation between infectious complications and rejection are of major clinical importance. Elevated PCT levels have been detected in patients following organ transplantation in a number of studies [4-6]. Mild PCT elevation can be a marker of surgical trauma. In some studies, PCT was evaluated for its sensitivity in distinguishing between acute rejection and infection [4,7]. Highly elevated PCT levels were described in patients having undergone immunosuppressive therapy. All patients were post-liver or post-kidney transplantation and were without the presence of systemic bacterial infection [6,8,9].

The aims of the present study were (a) to investigate serum levels of PCT and CRP in the perioperative and postoperative periods in patients undergoing orthotopic liver transplantation (OLTx) and receiving different perioperative inductive immunosuppressive therapy, (b) to address the possible molecular relationship between the liver transplantation with conjunctive immunotherapy and PCT production, and (c) toevaluate our results in patients undergoing liver resection without immunosuppressive therapy.

## Materials and methods

PCT and CRP levels were investigated in two groups of patients undergoing OLTx with different regimens of the immunosuppressive therapy and in one group of patients undergoing liver resection as a surgical control. In the first group of patients (*n *= 21), polyclonal antibodies against T lymphocytes were administered together with the anti-thymocyte globulin (ATG) (9 mg/kg) (Fresenius, Fresenius Biotech GmbH, Gräfelfing, Germany) and methylprednisolone 250 mg during the anhepatic phase. Afterward, patients received a combination of ATG (3 mg/kg up to postoperative day 3), cyclosporin A (7.5 mg/kg per day), and methylprednisolone. In the second group, 7 patients perioperatively received methylprednisolone 250 mg only. Subsequent therapy involved methylprednisolone and tacrolimus (0.1 mg/kg per day FK 506, tacrolimus [Fujimycin] immunosuppressive drug, macrolide antibiotic). The serum levels of PCT and CRP were measured before the induction of anesthesia, in the fourth and eighth hours after graft reperfusion, and continued daily to the fourth day after surgery.

The third group involved 12 patients undergoing liver resection. No infectious complications were observed during the early postoperative period (7 days). PCT and CRP levels were measured before the induction of anesthesia, immediately after the surgery, and then daily up to the fourth day after surgery.

Infection was defined as a clinical or microbiological infection. During the first 5 days, cultivation of urine and sputum as well as chest x-ray were carried out on a routine basis. If the body temperature exceeded 38°C, blood cultures were performed. The study was approved by the local ethics committee. All biological material was sampled upon informed consent.

### Blood samples

#### Procalcitonin and C-reactive protein measurements

Blood samples were collected as a routine test in accordance with the ethical guidelines of the hospital. All blood samples were stored at 4°C and were analyzed within 48 hours. PCT analyses were performed using an immunoluminometric assay (Lumitest-PCT; BRAHMS Aktiengesellschaft, Hennigsdorf, Germany) Analyses of CRP were performed using a fully automated turbidimetric assay.

### Statistical analysis

Summary data are expressed as median and range (minimum and maximum). Two-tailed tests were performed and considered significant at *p *values of less than 0.05. The Friedman nonparametric test was used to evaluate the time changes, and the Kruskal-Wallis analysis of variance was used to evaluate differences between groups.

## Results

**Figure 1:**
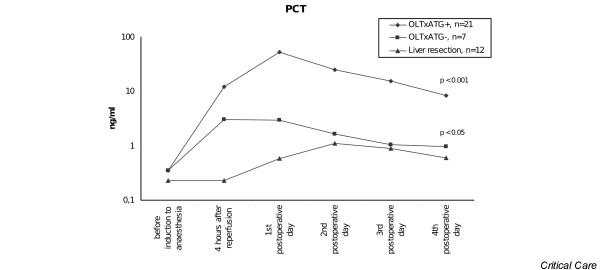
Procalcitonin (PCT) serum levels in patients after orthotopic liver transplantation (OLTx) with and without anti-thymocyte globulin (ATG) therapy and after liver resection.

PCT levels were significantly higher in liver transplantation patients with ATG therapy compared with patients without ATG therapy, with a significant difference being detectable 4 hours after the graft reperfusion (*p *< 0.001). PCT levels were significantly higher in both groups of patients undergoing liver transplant and receiving immunosuppressive therapy in comparison with patients with liver resection alone (*p *< 0.05) (Figure 1).

In patients with ATG therapy, the median PCT level 4 hours after reperfusion of the liver graft was 12.2 ng/mL (minimum 1.4 ng/mL, maximum 49.7 ng/mL), and the maximum levels were detected on the first postoperative day (median 53 ng/mL, minimum 7.9 ng/mL, maximum 249.1 ng/mL). Thereafter, values continuously decreased independently of further ATG administration. Elevated levels of PCT were also detected in patients undergoing liver transplant and immunosuppression without ATG therapy; however, peak levels reached only 13.9 ng/mL, again occurring on the first postoperative day (Table 1).

**Table 1:** Serum levels of PCT and CRP in patients after OLTx with and without ATG administration and after liver resection

OLTx with ATG, median (min-max) *n *= 21	Before induction to anesthesia	4 hours after reperfusion	8 hours after reperfusion	1st postoperative day	2nd postoperative day	3rd postoperative day	4th postoperative day
PCT, ng/mL	0.4 (0.1–1.24)	12.2 (1.4–49.7)	43.8 (8.64–206.8)	53.0 (7.9–249.1)	25.1 (3.4–187.1)	15.5 (2.2–82.8)	8.5 (1.16–23.0)
CRP, mg/L	8.0 (0.0–61.8)	19.8 (1.4–53.0)	28.5 (5.40–131.0)	78.0 (22.0–181.0)	59.0 (11.7–145.0)	25.0 (4.2–87.0)	16.0 (2.2–86.0)
							

OLTx without ATG administration, median (min-max) *n *= 7	Before induction to anesthesia	4 hours after reperfusion	8 hours after reperfusion	1st postoperative day	2nd postoperative day	3rd postoperative day	4th postoperative day

PCT, ng/mL	0.4 (0.1–1.1)	3.0 (0.8–3.8)	3.6 (1.1–11.6)	3.0 (1.4–13.9)	1.6 (1.0–8.8)	1.0 (0.6–4.4)	1.0 (0.2–3.1)
CRP, mg/L	12.0 (0.0–29.5)	27.4 (8.0–90.0)	54.0 (21.0–112.0)	61.0 (40.0–148.0)	51.0 (22.0–90.0)	28.0 (11.0–55.0)	23.0 (6.3–47.0)
							

Liver resection, median (min-max) *n *= 12	Before induction to anesthesia	After admission to intensive care unit		1st postoperative day	2nd postoperative day	3rd postoperative day	4th postoperative day

PCT, ng/mL	0.2 (0.1–0.7)	0.2 (0.1–0.8)		0.6 (0.1–2.5)	1.1 (0.3–3.6)	0.9 (0.2–3.6)	0.6 (0.2–1.8)
CRP, mg/L	4.8 (0.0–27.0)	4.4 (0.0–19.0)		29.0 6.0–80.0)	35.7 (12.0–142.0)	43.0 (7.0–173.0)	24.0 (5.0–144.0)

ATG, anti-thymocyte globulin; CRP, C-reactive protein; min-max, minimum to maximum; OLTx, orthotopic liver transplantation; PCT, procalcitonin.

In the group of patients undergoing liver resection, the median PCT on the first postoperative day was 0.6 ng/mL (minimum 0.1 ng/mL, maximum 2.5 ng/mL) and PCT levels peaked on the second postoperative day (median 1.1 ng/mL, minimum 0.3 ng/mL, maximum 3.6 ng/mL) and then decreased to the normal range (Figure 1).

**Figure 2:**
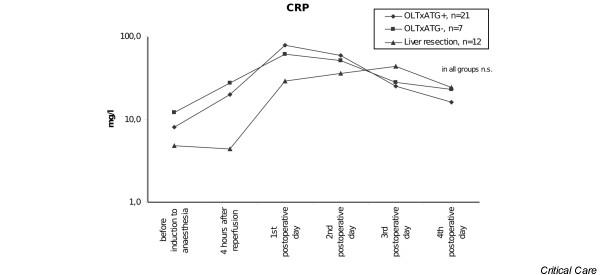
C-reactive protein (CRP) serum levels in patients after orthotopic liver transplantation (OLTx) with and without anti-thymocyte globulin (ATG) therapy and after liver resection. n.s., not significant.

CRP levels were elevated in patients with OLTx and immunosuppressive therapy with ATG on postoperative days 1 and 2 after reperfusion. The maximum levels were observed on the first postoperative day and then decreased. CRP levels also increased over the first 2 days postoperatively in patients with OLTx without ATG. In patients with liver resection, we found the maximum levels of CRP on the second postoperative day, with a subsequent decrease on the fourth postoperative day (Table 1). In contrast to PCT, no differences were found in CRP levels in any of the groups of patients (Figure 2). None of the patients in this study had any evidence of rejection over the first month after transplantation and there were no septic complications during this period either.

## Discussion

The role of PCT under physiological conditions and in sepsis has not been fully elucidated yet. Experimental and clinical studies imply that PCT might act as a toxic factor in severe bacterial inflammation [10]. Regarding its place of origin, PCT is a rather ubiquitous molecule [11-13]. The absence of PCT production in the model of hepatectomized baboons suggests a primary role for the liver as a source of PCT production during endotoxin shock [14].

Our results seem to illustrate the link between the type of immunosuppressive drug and induction of PCT production. Transient elevation of PCT due to the immunosuppressive therapy in kidney or liver transplant patients has been described in other studies [5,6,8,9]. In those studies, as well as in our cohort of patients, no systemic bacterial infection has been detected. Interestingly, in each group of patients, a different immunosuppressive therapy was used: ATG, anti-CD3 monoclonal antibody, and systemic corticosteroids.

What is important from the clinical point of view? If there is a systemic bacterial infection present in a patient, one of the most important issues in the monitoring of PCT is its dynamics. In our study, in both groups of patients receiving immunosuppressive therapy, an elevated level of PCT was found, especially in the group of ATG-treated patients. The PCT levels reached their peak values on the first postoperative day and ceased thereafter in all patients. None of the patients displayed any sign of systemic bacterial infection. These results correlate very well with the findings of Kuse and colleagues [6,8] in a group of patients after liver transplant. Similar results have been found by Sabat and colleagues [9] in patients after liver transplant. The study of Fazakas and colleagues [15] documented only a mild elevation of PCT levels immediately after graft reperfusion in patients after liver transplantation without OKT3 (monoclonal antibody that specifically reacts with the T cell receptor-CD3 complex on the surface of circulating human T cells) or ATG therapy. Thus, it is noticeable that very high levels of PCT (in the range of hundreds of nanograms), even if elevated only transiently, are not connected with the presence of systemic bacterial infection.

The regulatory processes connected with such high levels of PCT are complicated to address, and, at present, only hypotheses are available. Kuse and colleagues [6,8] speculated that systemic cytokine release induced by OKT3/ATG administration could lead to increased enteral permeability with endotoxin translocation causing the PCT increase. In contrast to the findings of this study are those of Redl and colleagues [16], who assessed the correlation of PCT levels and tumor necrosis factor in an *Escherichia coli *model in baboons. They did not find any correlation between PCT and tumor necrosis factor levels. Our results support the study of Sabat and colleagues [9] with patients after kidney transplantation, in which the highest PCT levels were found in patients with ATG therapy. What is the reason for this? Polyclonal ATG is produced by immunization of rabbits with the human Jurkat T cell line. One of the molecules expressed in the Jurkat T cell line is intracellular adhesion molecule-1 (CD54), which is involved in the process of inflammation, and its expression is induced by ischemia. The liver and mononuclear cells could represent a potential source of PCT production triggered by ATG therapy and cold ischemia during perioperative management. Concerning these findings, we suspect that, in the case of polyclonal antibodies, more binding epitopes and more targets for the initiation of the process of inflammation are present.

## Conclusion

The type of immunosuppressive therapy influences PCT serum levels in patients after OLTx. Administration of pan-T-cell antibodies to patients with OLTx is associated with a significant increase in serum PCT levels, with peak values on the first postoperative day. PCT rises in the absence of any clinical and microbiological evidence of sepsis. Further studies are needed to elucidate the mechanisms responsible for the PCT production.

## Key messages

• The type of immunosuppressive therapy influences procalcitonin (PCT) serum levels in liver transplant patients.

• Administration of anti-thymocyte globulin (ATG) is a powerful stimulus for PCT synthesis and release.

• The transient high PCT values in orthotopic liver transplantation patients with and without ATG treatment are not caused by bacterial infection.

• There is still limited knowledge on the mechanisms of PCT synthesis and release.

## Abbreviations

ATG = anti-thymocyte globulin (polyclonal antibodies against human T cells); CRP = C-reactive protein; OKT3 = monoclonal antibody that specifically reacts with the T cell receptor-CD3 complex on the surface of circulating human T cells; OLTx = orthotopic liver transplantation; PCT = procalcitonin.

## Competing interests

The authors declare that they have no competing interests.

## Authors' contributions

RZ helped conceive, design, and carry out the study and shares responsibility for its outline. MP helped conceive, design, and carry out the study, shares responsibility for its outline, and performed laboratory analyses. TT and EK performed the literature search, identified the relevant studies to be included in the analysis, and compiled the data for the study. All authors contributed to the writing of the manuscript and approved of its final version.
